# The dark side of the spoon - glucose, ketones and COVID-19: a possible role for ketogenic diet?

**DOI:** 10.1186/s12967-020-02600-9

**Published:** 2020-11-20

**Authors:** Antonio Paoli, Stefania Gorini, Massimiliano Caprio

**Affiliations:** 1 Department of Biomedical Sciences, University of Padua, Padua, Italy; 2 Laboratory of Cardiovascular Endocrinology, IRCCS San Raffaele Pisana, Via di Val Cannuta, 247, 00166 Rome, Italy; 3 Department of Human Sciences and Promotion of the Quality of Life, San Raffaele Roma Open University, Via di Val Cannuta, 247, 00166 Rome, Italy

**Keywords:** Sars-CoV-2, VLCKD, Obesity, Diabetes, Hyperglycaemia, Inflammation, Metabolic rehabilitation

## Abstract

The novel coronavirus disease (COVID-19) is posing a serious challenge to the health-care systems worldwide, with an enormous impact on health conditions and loss of lives. Notably, obesity and its related comorbidities are strictly related with worse clinical outcomes of COVID-19 disease. Recently, there is a growing interest in the clinical use of ketogenic diets (KDs), particularly in the context of severe obesity with related metabolic complications. KDs have been proven effective for a rapid reduction of fat mass, preserving lean mass and providing an adequate nutritional status. In particular, the physiological increase in plasma levels of ketone bodies exerts important anti-inflammatory and immunomodulating effects, which may reveal as precious tools to prevent infection and potential adverse outcomes of COVID-19 disease. We discuss here the importance of KDs for a rapid reduction of several critical risk factors for COVID-19, such as obesity, type 2 diabetes and hypertension, based on the known effects of ketone bodies on inflammation, immunity, metabolic profile and cardiovascular function. We do believe that a rapid reduction of all modifiable risk factors, especially obesity with its metabolic complications, should be a pillar of public health policies and interventions, in view of future waves of SARS-CoV-2 infection.

## Introduction

Data from World Health Organization (WHO) indicate that seasonal influenza causes about 3 to 5 million cases of severe illness, 290,000 to 650,000 deaths from respiratory causes and 99,000–200,000 deaths from lower respiratory tract infections [1]. In general, acute respiratory infections are one of the leading causes of morbidity around the world and, more specifically, lower respiratory tract infections are the leading infectious cause of death and the fifth-leading cause of death overall.

In consideration of the relative low effectiveness of influenza vaccination, public health practices have been implemented by health authorities in order to limit the spread of respiratory viruses. Common suggested health practices are related to peoples’ behaviors as hand washing, use of face masks and eye protection, social distancing, and were encouraged during the pandemic spread of Coronavirus 2019 disease (COVID-19), caused by a virus (SARS-CoV-2) for which there is not yet a vaccine [2], nor effective pharmacological treatments, so far.

## Risk factors for COVID-19: role of lifestyle behaviors

Despite the current mortality rate is 2.3% [3], the emergence of large number of infected patients within a short period of time determined relevant difficulties in health-care system of several countries, with an enormous loss of lives. Among risk factors for SARS-CoV-2, numerous studies identified a specific association of COVID-19 fatality with advanced chronological age [3] and presence of comorbidities, particularly diabetes, hypertension, obesity and chronic kidney disease [4]. Some of these diseases are related to lifestyle behavior, thus it should be mandatory to act on the aforementioned risk factors to improve patients’ outcomes and to reduce health’s impact of possible future new outbreaks [5].

It is worth to mention that also the increase of sedentarism happened during the recent lockdown measures led to a negative impact on general health condition. The sedentarism due to shelter in place policy enforced in several countries during COVID-19 pandemic, displayed deleterious effects on skeletal muscle metabolism, inducing insulin resistance, fat deposition and low-grade systemic inflammation, with an overall worsening of metabolic parameters, glucose control and inflammatory status. In this regard, sarcopenic obesity, characterized by the coexistence of excessive adiposity and low muscle mass, is strongly associated with increased cardiovascular risk, insulin resistance and low-grade inflammation; therefore, it represents, together with inadequacy of nutritional status, a condition associated with increased susceptibility to viral infection [6]. Importantly, critically ill COVID-19 patients with obesity and malnutrition showed worse outcomes than obese patients without malnutrition [7].

During the lockdown, lifestyle and eating habits of a great part of the worldwide population have dramatically changed. The most important nutritional advice was to reduce the consumption of junk food and to prefer food items with antioxidant and anti-inflammatory properties, with the potential to positively affect the immune system [8, 9]. It is known that unhealthy nutritional habits, rich in fat and carbohydrates, are associated with obesity and sleep disturbances. Importantly, a marked decrease in sleep quality and an associated increase in body mass index has been described during the quarantine [10]. Moreover, the decreased amount of physical activity during quarantine has affected significantly population’s general health [6, 11, 12]. Therefore, there is the real risk that a higher number of patients with obesity and poor blood glucose control will be exposed to novel waves of Sars-Cov2 infection.

## Obesity and hyperglycemia: two *villains* on COVID-19 stage

Obesity represents one of the recognized prognostic factor for requirement of intensive care and high risk of death during SARS-CoV-2 infection [13]. In keeping with this, an important shift of severe COVID-19 disease to younger age has been clearly documented with obesity [14]. Obesity affects several critical functions, characterizing an increased vulnerability towards COVID-19 adverse outcomes [15]. Obesity state restricts ventilation by disrupting diaphragm excursion, alters immune responses to viral infection [16], determines a chronic, low-grade inflammation, and worsens glucose tolerance and oxidative stress with adverse effects on cardiovascular function [17]. Importantly, obese patients experience a more severe COVID-19 syndrome, since obesity is characterized by an altered hemostatic balance with increased coagulation and defective fibrinolysis, which results in a pro-thrombotic state [18]. Furthermore, the co-existence of obesity and metabolic-associated fatty liver disease (MAFLD) determines a ~ 6-fold increased risk of severe outcome of COVID-19, independently of age, sex, smoking, diabetes, hypertension and dyslipidemia [19].

Notably, a recent report showed that adipose tissue expresses very high levels of transcripts for ACE2 [20], an enzyme attached to the outer surface of pneumocytes, which is used by coronaviruses to enter and infect cells, raising the question whether adipose tissue may represent a reservoir of SARS-CoV-2, and a strategical site to amplify the cytokines cascade triggered by viral infection [21].

The global epidemic of COVID-19 has also determined important implications on the therapy of common metabolic disorders such as type 2 diabetes (T2D), since two coronavirus receptor proteins, dipeptidyl peptidase-4 (DPP4) and angiotensin-converting enzyme 2 (ACE2) are well established transducers of metabolic signals controlling glucose homeostasis, together with other pathways involved in the regulation of cardiovascular physiology, inflammation and renal activity [22]. Glucose-lowering agents like DPP4 inhibitors, widely used in T2D therapy, can modify the biological activities of multiple immunomodulatory substrates [23]. ACE2 is the entry receptor recognized by the spike (S) protein of SARS-CoV-2, and is known to be expressed in several different tissues including the lung, the kidney tubules, the heart, the luminal surface of the small intestine, adipose tissue and blood vessels [24]. Urinary ACE2 protein concentration and its enzymatic activity are increased in subjects with both type 1 diabetes (T1D) [25] and T2D [26]. Whether hyperglycemia can regulate ACE2 expression in human tissues has not been sufficiently established yet, but it is known that in mouse models of diabetes, ACE2 is overexpressed in the lung, kidney, and heart [27]. In addition, several medications for the treatment of hypertension such as ACE inhibitors (ACEi) and angiotensin receptor blockers (ARBs), are known to upregulate ACE2 expression [28]. Thus, due to increased ACE2 receptor expression in multiple tissues of diabetic patients, it has been argued that the severity of COVID-19 might potentially be exacerbated [29]. Nevertheless, in consideration of the well-established organ protective effects of renin angiotensin aldosterone system (RAAS)-inhibitors, several authoritative scientific societies recommended against the discontinuation of these drugs in patients with high risk of COVID-19 infection [30]. However, the possibility to avoid or interrupt potentially harmful pharmacological therapies with a lifestyle intervention should be carefully taken into consideration.

Diabetes is associated with an increased risk of severe viral respiratory tract infections, including H1N1 influenza, given that elevated glucose levels can also suppress anti-viral responses [31]. In China, a study on more than 500 subjects hospitalized with SARS-CoV-2 revealed that high fasting glucose levels determined increased rates of death [32].

Moreover high glucose plasma levels and diabetes were independent predictors of mortality in patients with SARS [33] and during the 2009 H1N1 pandemic, diabetes was shown to increase the severity of infection [31]. In accordance, influenza and MERS-CoV-infected diabetic mice showed increased illness gravity [34, 35].

In a study conducted on 5700 patients affected by COVID-19 and hospitalized in the New York City area, diabetic patients were more likely to have received invasive mechanical ventilation compared with non-diabetic ones [36] suggesting that individuals with diabetes may display impaired alveolar function. Importantly in diabetic animal models structural changes in the lung were observed, such as collapsed alveolar epithelium and elevated vascular permeability [37]. Moreover, endothelial capillary basal lamina and alveolar epithelium are thicker in diabetic patients compared to non-diabetic individuals [38]. Notably, a recent work by Iacobellis and colleagues showed that hyperglycemia at admission was the best predictor of radiographic imaging of acute respiratory distress syndrome (ARDS), independently of the medical history of diabetes. Acute hyperglycemia may lead to an abnormal inflammatory and immune response contributing to the development and progression of the radiographic findings of ARDS in patients with COVID-19 [39]. In addition, structure–function studies suggest that the spike protein of SARS-CoV-2 is highly glycosylated [40] and it has been hypothesized that high concentration of glycosylated SARS-CoV-2 viral particles and glycosylated ACE2 in the lung epithelium, due to hyperglycemia, may influence the susceptibility to COVID-19 infection and its subsequent severity [41]. As a matter of fact, higher glucose plasma levels can increase glucose concentration in airway secretions, and pulmonary epithelial cells exposure to high glucose concentrations is known to enhance influenza virus infection and replication [42, 43]. A recent report provided evidence that elevated glucose levels are strictly involved in viral replication: interestingly, SARS-CoV-2 infection increases mitochondrial ROS production, which induces stabilization of hypoxia-inducible factor-1α (HIF-1α). This, in turn, shifts the metabolic features of monocytes/macrophages into highly glycolytic, leading to an exaggerated SARS-CoV-2 replication rate [44]. In keeping with this, a recent study in vitro showed that pharmacological inhibition of glycolysis reduced SARS-CoV-2 viral replication in a human colon epithelial carcinoma cell line (Caco-2 cells). Therefore, a reduction of aerobic glycolysis could be important as a metabolic therapy aimed at controlling viral replication [45].

For all these reasons, hyperglycemia and high glycemic variability should be adequately prevented in order to improve the outcomes of COVID-19 patients. It is hence clear that well-controlled blood glucose levels will correlate with lower risk of infection and/or better disease resolution. In consideration of potentially adverse effects of commonly used drugs for type 2 diabetes and its comorbidities, a nutritional approach lowering blood glucose concentrations should be considered as a first option, in order to prevent or reduce Sars-Cov-2 infection risk and related complications.

## Inflammation: our mutual friend, which may become harmful

It is well established that the aberrant release of pro-inflammatory cytokines and chemokines, induced by SARS-CoV-2 infection, is central for the fatal outcomes of COVID-19 syndrome [46]. A severe progression of COVID-19 disease is determined by a tardive interferon gamma response with a prolonged inflammatory state and lower Treg, NK, and both CD4+ and CD8+ cells counts [47, 48]. It is exhaustively documented that hyperglycemia may worsen the inflammatory response. As a matter of fact, NK cells activity is reduced and pro-inflammatory M1 macrophages are elevated in diabetic patients [49, 50]. Furthermore, individuals with diabetes display a chronic low-level pro-inflammatory state, with a well-established Th17/Treg and Th1/Th2 imbalance [51]. High glucose levels amplify cytokine production in monocytes through an increase in mitochondrial ROS [44]. It is hence likely that dysregulated immune cell populations and activity observed in diabetic patients represent important risk factors and determine worsening of the inflammatory response during SARS-CoV2 infection.

Obesity and, more in general, excess adipose tissue are characterized by a low-grade chronic inflammation state. Even though the precise obesity-inflammation mechanism is still not well known, some clues indicate as responsible gut-derived molecules, diet-derived metabolites (such as free fatty acids), intrinsic factors related to the enlargement of the adipocytes such as hypoxia, mechanotransduction, cells death [52] and altered secretion of adipokines and cytokines. Monocyte/macrophage recruitment, which is functional for the removal of cellular debris due to adipose cell death, amplifies these mechanisms [53]. Chronic low-grade inflammation should be carefully considered in order to understand the risks of obesity and associated diseases, since several key inflammatory markers are strictly linked with an increased risk of adverse outcomes in obesity-associated comorbidities, such as cardiovascular disease and T2D. There is a large body of evidence indicating a positive correlation between C-reactive protein, known as a marker of systemic inflammation, and body composition [54]. Moreover plasminogen-activator inhibitor [55], erythrocyte sedimentation rate [56] and key inflammatory cytokines [57, 58], show a similar positive association in obese individuals, further supporting the strict connections between inflammation and obesity [59–61].

## Ketogenic diet as lifestyle behavior approach against COVID-19

Among lifestyle behaviors it is surprising that nutritional advice are still poorly considered in public health discussions about the prevention or reduction of Sars-Cov-2 infection risk and related complications. Thus, a call to action is needed, in order to promote proper nutrition strategies to improve the immune response and the potential clinical outcomes towards COVID-19.

Recently, Soliman et al. proposed a combination of intermittent fasting and supplementation in medium-chain triglycerides as potential prophylactic strategies or adjuvant therapy to tackle SARS-CoV-2 infection, by means of a change in the host metabolic state from a carbohydrate-dependent glycolytic to a fat-dependent ketogenic state, aimed to alter viral replication [62]. Such metabolic shift causes an increased resistance to mitochondrial stress, an improvement in antioxidant defenses, an augmented autophagy and DNA repair, and a decreased insulin secretion [63]. In this context, ketogenic diets represent a nutritional approach with intriguing theoretical bases for improving the immunological response to Sars-CoV-2 infection in high-risk populations [64].

### Ketogenic diets

Ketogenic diets (KDs) are high-fat, low carbohydrate diets and have been primarily used to treat epilepsy in children since the 1920s [65]. Interestingly, their use has been adapted to face different pathologic conditions (severe obesity, metabolic diseases, migraine, cancer, etc.), by changing macronutrients composition and energy content. In this context, the importance of KDs in the treatment of obesity and its associated comorbidities (T2D, dyslipidemia, insulin resistance, inflammation) has recently emerged [66, 67]. In particular, very low calorie ketogenic diets (VLCKDs), characterized by a marked restriction of carbohydrate intake, usually lower than 30 g/day, with a relative increase in the proportions of fat and protein and a total daily energy intake < 800 kcal, represent a highly effective nutritional strategy in patients who need a rapid weight loss over a short term period, such as individuals with moderate to severe obesity and associated cardiovascular risk factors, leading to a marked improvement in insulin resistance, glucose and blood pressure control [66, 67]. Weight loss obtained with VLCKD is mostly secondary to fat mass loss, whereas lean mass and adequate nutritional status is preserved [68]. Importantly, KD has been proven to be an effective and rapid treatment for MAFLD, markedly decreasing liver fat content and hepatic insulin resistance only after 6 days of treatment [69]. Indeed, KDs require a strict medical supervision and therapeutic compliance, together with proper micronutrients and vitamins supplementation. For this reason, contraindications to its utilization should be carefully taken into consideration [67].

There is a large body of evidence demonstrating the efficacy of VLCKD in blood glucose control [52]. As a matter of fact, the very low intake in carbohydrates in this nutritional protocol dampens large spikes in blood sugar, thereby improving glycaemic variability. In obese patients with T2D short term exposure to VLCKD determines a marked improvement in β-cell function, at a higher extent than what could be explained by the weight loss obtained. The drastic reduction in carbohydrate intake is associated with a significant suppression of hepatic glucose production, due to a marked improvement in hepatic insulin sensitivity [70]. An enhanced insulin response has also been described after a short exposure to a VLCKD regimen, with a recovery of insulin response to a hyperglycemic challenge [71]. For all these reasons, VLCKD could be part of a multidisciplinary strategy for metabolic rehabilitation in patients with diabesity.

KDs exert most of their therapeutic effects by increasing plasma levels of ketone bodies [AcetoAcetate and Beta-hydroxybutyrate (ßOHB)] and decreasing blood glucose. In humans, basal plasma levels of ßOHB are in the low micromolar range, and reach stable levels around 1 mM during a KD. Beyond its role as energy fuel, ßOHB exerts pleiotropic and heterogeneous effects in cellular physiology, inducing the expression of genes that curtail oxidative stress and displaying remarkable immuno-modulating, anti-catabolic [72] and anti-inflammatory [73] effects by different mechanisms in multiple tissues, as recently discussed in an exhaustive review article by Stubbs et al. [74]. Moreover, the well-known blood glucose lowering effect of KDs may help to contrast virus infection [44]. Finally, VLCKD has been found effective in blood pressure lowering, due to the increased natriuresis which is associated with ketone bodies urinary excretion [75]. In consideration of the reduced amount of physical activity caused by quarantine and the general changes of daily habits in the populations, physical exercise seems to be a potentiation factor of the aforementioned favorable effects of KDs, such as the improvement of glycaemic control [76], body composition [77], liver fat content [78], and in general metabolic health [79].

## Potential preventive effects of ketogenic diet on SARS-CoV-2 infection

Ketogenic diet may play a role modulating both innate and adaptive immune cells, which synergistically protect the host against pathogens’ assaults.

### Innate cell-mediated immunity

**Fig. 1:**
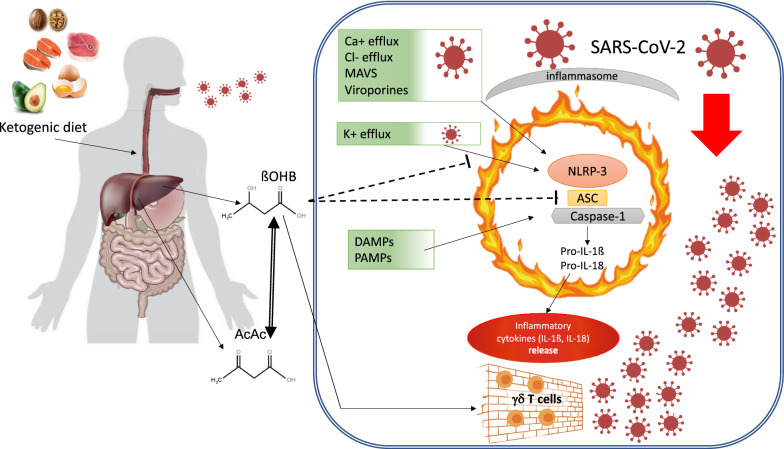
Ketone bodies and NLRP3/inflammasome activation. Protective effects of ketogenic diet and ßOHB on risk conditions associated with serious COVID-19 disease. ßOHB: Beta-hydroxybutyrate, AcAc: AcetoAcetate, MAVS: mitochondrial antiviral signalling protein, LPS: lipopolysaccharide, NLP3: NOD-, LRR- and pyrin domain-containing protein 3, ASC: Adaptor apoptosis associated Speck-like protein containing a Caspase Recruitment Domain (CARD), Pro-IL-1ß: pro-interleukin 1 beta, Pro-IL-18: pro-interleukin 18, DAMPs: damage-associated molecular patterns; PAMPs: pathogen-associated molecular patterns

Innate immune cells are firstly triggered by viral antigens through the activation of pattern recognition receptors (PRRs), in order to inhibit viral replication and modulate the adaptive immunity [80]. In this context, the NLRP3/inflammasome is an important innate immunity sensor, mediating virus-induced inflammation through the induction of Interleukine-1β (IL-1β) and Interleukine-18 (IL-18) secretion [81]. The pattern recognition receptor NLRP3 is a nucleotide oligomerization domain (Nod)-like receptor (NLR) that recognizes both damage-associated molecular patterns (DAMPs), such as toxins, ATP, excess of glucose, cholesterol crystals, and pathogen-associated molecular patterns (PAMPs), such as viral and bacterial molecules. For instance, RNA viruses can activate NLRP3 through mitochondrial antiviral signalling protein (MAVS) on the mitochondrial outer membrane. Activated NLRP3 promotes the formation of the inflammasome complex interacting with the adaptor protein ASC (apoptosis-associated speck-like protein containing C-terminal caspase recruitment domain [CARD]), which, in turn, triggers the activation of the zymogen procaspase-1 into caspase-1. Finally, the inflammatory caspase-1 converts the inactive pro-Interleukine-1β (pro-IL-1β) and pro-Interleukine-18 (pro-IL-18) into their corresponding active proinflammatory cytokines [82] (Fig. 1).

The NLRP3/inflammasome activation due to a viral infection has been documented for influenza A virus (IAV), encephalomyocarditis virus (EMCV), hepatitis C virus (HCV), and SARS-CoV, and seems to be mediated also by viral proteins known as viroporins, specific molecules [83] which assemble into homo-oligomers and form hydrophilic pores across the cytosolic organelle membranes, thereby increasing Na+, K+, and Ca2+ flux. Increased intracellular Ca2+ concentration and reduced intracellular K + levels represent important triggering signals for NLRP3/inflammasome activation and the subsequent massive secretion of proinflammatory cytokines [84]. All coronaviruses known so far, including the new spread SARS-CoV2, are able to encode for viroporines E and 3a and their expression is functional to the activation of NLRP3/inflammasome in COVID-19 disease [83].

There is growing evidence that ßOHB inhibits NLRP3/inflammasome activation. The favorable effects of KD on inflammatory cytokines in humans [85], in animal and cellular models [86] are well established. ßOHB is able to act on a central common signaling pathway, specific to the NLRP3/inflammasome, in response to many different pro-inflammatory stimuli. More specifically, ßOHB inhibits NLRP3/inflammasome activation through the reduction of K+ efflux from macrophages and the inhibition of the inflammasome assembly (Fig. 1). Consistent with these observations, ßOHB-dependent inhibition of IL-1β and IL-18 secretion in human monocytes has been documented [73].

In consideration of the role of inflammasome activation in triggering the systemic inflammatory cascade observed in COVID-19 patients [46], approaches based on increasing plasma ßOHB, such as KDs, should be taken into account to prevent the development or the progression of the cytokine storm syndrome.

Interestingly, a recent hypothesis paper underlined the importance of a drastic reduction of glucose oral supply, in order to reduce macrophage M1 polarization in the early stages of inflammation [64]. In fact, the M1 phenotype, whose activation is linked to the cytokine storm syndrome [87], is strictly dependent upon aerobic glycolysis, which is known to be reduced by a drastic reduction in glucose uptake, as it occurs during a KD. On the other hand, KDs could sustain the metabolism of anti-inflammatory M2 macrophages, which abundantly express OXPHOS enzymes through the continuous supply of free fatty acids [64].

### Adaptive cell-mediated immunity

T lymphocytes recognize specific ligands by Tcell receptors (TCR), which are specialized in antigen recognition. In most species, the vast majority of T cells TCR is composed by an α and a β chain, and a minor Tcell population expresses a TCR characterized by γ and δ chains. In humans and mice most of T cells (> 90%) in peripheral blood and lymphoid organs express the TCR α/β chain and only a minority of T cells (< 10%) express the TCR γ/δ. Interestingly, in mice, γδ T cells are the most abundant Tcell population in epithelia and mucosa [88]. The epithelial layers display a peculiar immune system and resident T lymphocytes, which are in close contact with the epithelial cells. In humans, γδ T cells are enriched in skin and mucosa, suggesting a specific function for γδ T cells in mucosa layers [88]. Therefore, these cells may play an important role in viral infection surveillance and response in mucosal inner layers of the respiratory trait.

A recent study by Goldberg et al. showed that immunocompetent mice exposed to intranasal challenge of influenza A virus (IAV), displayed better survival when their γδ T cell population were increased in the lung, determining an improvement of barrier function and anti-viral response [89]. More specifically, mice underwent γδ T cell expansion by means of a ketogenic diet for 7 days, and displayed a better blood O_2_ saturation compared to control chow-fed mice, with an increased secretory function, mucus production in the airways, and IL-17 production, thereby mediating anti-viral defense and tissue repair through regulatory T cells (Treg) activation, a cell population which is known to be reduced during the COVID-19 cytokine storm [47].

Notably, γδ T cells expansion was specifically promoted by KD, since pharmacological increase of ßOHB failed to induce this phenotype [89], and only endogenous ketone bodies—not the exogenous ketone precursor 1,3-butanediol—were able to protect mice against influenza infection. Importantly, γδ T cells can expand in response to IAV and kill IAV-infected airway cells also in humans [90]. Therefore, KD could represent a valuable option in order to physiologically increase ßOHB levels, and optimize adaptive immune cells to prevent Sars-Cov2 infection.

A recent observation showed that γδ T cells are also expressed in adipose tissue [91], where they increase IL-17 production, thereby promoting the expansion of Treg cells function, with immuno-modulatory and anti-inflammatory properties.

It is therefore tempting to speculate a key role for γδ T cells in maintaining barrier integrity against Sars-Cov2 infection in the lung, as well as in adipose tissue, where meta-inflammation could enhance the cytokine reaction in response to viral infection. KD may represent a valid approach to specifically sustain these protective mechanisms (Fig. 1).

## Conclusions

There are multiple mechanisms through which ketone bodies might impact severe viral infections such as COVID-19 disease. A recent review article exhaustively summarizes this concept, proposing the administration of exogenous ketones to critical patients in order to target respiratory viruses complications as a possible therapy [74].

We believe that KD-induced increase in endogenous ketone bodies could represent a more valuable strategy to prevent Sars-Cov2 infection and adverse outcomes in obese patients, particularly in the current context of a prolonged pandemic emergency. Indeed, prevention and/or correction of all risk conditions associated with serious COVID-19 disease (obesity, hyperglycemia, high glycemic variability, insulin resistance, hypertension) is mandatory, in consideration of new waves of infections, in the absence of effective pharmacological therapies and vaccination. This could be obtained with a nutritional strategy aimed to induce fat mass loss, to reduce chronic inflammation, hepatic and systemic insulin resistance, and to improve nutritional status, cardiovascular health, immune response, glucose homeostasis and blood pressure control.

Finally, the adoption of a well-structured and personalized KD regimen could help a progressive nutritional education and rehabilitation in obese patients, providing an effective tool to modify lifestyle behavior, supporting a long-term control of body weight, and favoring a reduction in all associated risk factors for potentially severe complications related to Sars-Cov2 infection. Well-designed multicentric studies on the actual incidence of severe COVID-19 disease among obese patients who followed or not a structured protocol of KD, could be helpful to confirm such hypothesis.

During this difficult pandemic era, the adoption of lifestyle preventive measures is mandatory, and should be carefully implemented.

## Data Availability

Not applicable
